# Amplicon-Metagenomic Analysis of Fungi from Antarctic Terrestrial Habitats

**DOI:** 10.3389/fmicb.2017.02235

**Published:** 2017-11-14

**Authors:** Marcelo Baeza, Salvador Barahona, Jennifer Alcaíno, Víctor Cifuentes

**Affiliations:** Laboratorio de Genética, Departamento de Ciencias Ecológicas, Facultad de Ciencias, Universidad de Chile, Santiago, Chile

**Keywords:** Antarctic microeukaryotes, amplicon-metagenome, Antarctic fungi, Antarctic diatoms, cold-terrestrial microeukaryotes

## Abstract

In cold environments such as polar regions, microorganisms play important ecological roles, and most of our knowledge about them comes from studies of cultivable microorganisms. Metagenomic technologies are powerful tools that can give a more comprehensive assessment of microbial communities, and the amplification of rDNA followed by next-generation sequencing has given good results in studies aimed particularly at environmental microorganisms. Culture-independent studies of microbiota in terrestrial habitats of Antarctica, which is considered the driest, coldest climate on Earth, are increasing and indicate that micro-diversity is much higher than previously thought. In this work, the microbial diversity of terrestrial habitats including eight islands of the South Shetland Archipelago, two islands on the Antarctic Peninsula and Union Glacier, was studied by amplicon-metagenome analysis. Molecular analysis of the studied localities clustered together the islands of the South Shetland Archipelago, except Greenwich Island, and separated them from the Litchfield and Lagotellerie islands and Union Glacier, which is in agreement with the latitudinal difference among them. Among fungi, 87 genera and 123 species were found, of which species belonging to 37 fungal genera not previously cultivated from Antarctica were detected. Phylogenetic analysis, including the closest BLAST-hit sequences, clustered fungi in 11 classes being the most represented Lecanoromycetes and Eurotiomycetes.

## Introduction

The majority of the biosphere on Earth is classified as a cold environment, where microorganisms representing the three domains of life, Bacteria, Archaea and Eukarya, have key ecological roles (Cantrell et al., 2011; Margesin and Miteva, 2011; Buzzini et al., 2012). These cold-adapted microorganisms have attracted scientific interest mainly for their potential application in diverse productive areas, such as food, biofuel and textile industries and cold environments such as polar regions have become attractive for bioprospecting for new genes and bioactive compounds with potential commercial uses (Gerday et al., 2000; Margesin et al., 2007; de Pascale et al., 2012). Most studies of microbiota in cold environments focused on cultivable microorganisms (Cary et al., 2010; de Pascale et al., 2012; Gugliandolo et al., 2016), but it is estimated that only approximately 17% of fungi can be cultivated using current methodologies (Hawksworth, 2001). Metagenomic technologies have become a powerful tool for more comprehensive assessment of microbial communities in the soils of extreme environments (Cowan et al., 2015). Amplicon-metagenome analysis has given good results when studying some microorganisms by amplifying a target rDNA followed by next-generation sequencing (NGS). With advances in NGS technologies, greater sequencing depth can be obtained at relatively low cost. The amplification of internal transcribed spacers (ITS) and the D1D2 domain of the large ribosomal subunit through polymerase chain reaction (PCR) amplification using universal primers (Kurtzman and Robnett, 1998) coupled with high-throughput sequencing (Lindahl et al., 2013) has been successfully used as a culture-independent approach to study soil fungal communities. Moreover, the incorporation of barcode sequences to primers allows high-throughput analysis of multiple samples (Tonge et al., 2014; Tedersoo et al., 2015).

Although the Antarctic continent is considered as a dry and cold climate, the regions closer to the sea are free of snow and ice, submitted to rapid cycles of freeze/thaw, and may receive significant quantities of organic material from marine animals (Holdgate, 1977; Ruisi et al., 2007). Studies of Antarctica microbiota are increasing, and studies applying culture-independent methodologies suggest that micro-diversity in Antarctic terrestrial habitats is much higher than previously thought (Cowan, 2014). Thousands of species were found in metagenomic and metatranscriptomic analyses of subglacial ice from the Vostok Lake in Antarctica; among them 6% corresponded to eukarya, predominantly closer to fungal species from the Ascomycota and Basidiomycota phyla (Rogers et al., 2013). Sequences mainly belonging to Proteobacteria and Actinobacteria were found in metagenomic analysis of soils from the Mars Oasis on Alexander Island (Pearce et al., 2012), and sequences close to the Flavobacterium genus were predominant in surface snow close to Russian stations in Eastern Antarctica (Lopatina et al., 2016). Furthermore, new genes from Antarctica have been found through metagenomic and functional analysis, such as a new cytosolic esterase from the α/β hydrolase family 6 (Berlemont et al., 2013); a methylthioadenosine phosphorylase gene, which was proposed as a novel reporter gene for promoter trapping (Bartasun et al., 2012); bacterial hydrocarbon-degrading genes (Luz et al., 2010); genes encoding proteins that functionally respond to environmental stress such as exopolysaccharides, cold shock proteins, and membrane modifications (Varin et al., 2012); and genes encoding enzymes with potential in industrial applications such as lipase/esterase, amylase, protease and cellulase (Berlemont et al., 2011, 2013).

The microfungal diversity in the terrestrial habitats of eight islands of the South Shetland Archipelago, two islands located on the Antarctic Peninsula and from Union Glacier, was studied in this work through amplicon-metagenome analysis. Among our findings, the most represented fungal classes among all Antarctic localities were Lecanoromycetes and Eurotiomycetes, and species belonging to 37 fungal genera, which have not been previously cultivated from Antarctica were found, such as including *Circinaria*, *Rhizoplaca*, and *Psoroma*. A correlation between molecular similarity and the geographic latitude of the studied localities was observed, grouping the islands of the South Shetland archipelago (latitude S61-S63) separately from localities in the Antarctic peninsula: Litchfield (S64-S65) and Lagotellerie (latitude S67-S68) islands, and Union Glacier (S79-S80).

## Materials and Methods

### Study Sites

**FIGURE 1:**
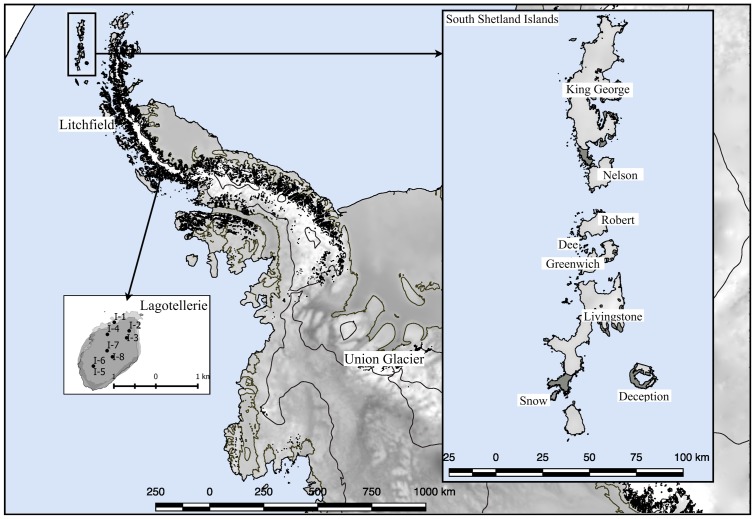
Sampling site locations. Sampling locations used in this work. Detail of principal sampling sites (marked as I-#) at Lagotellerie Island in the Antarctic Peninsula is shown. For details of sampling sites from other locations, see the following references (Carrasco et al., 2012; Barahona et al., 2016; Troncoso et al., 2017).

Several expeditions to Antarctica were performed to collect soil samples in sterile 50 ml plastic tubes; the tubes were sealed and shipped at -20°C to the Genetics Laboratory of the Faculty of Science at the Universidad de Chile. Once samples arrived at the laboratory, they were maintained at -80°C until processing. The collection dates were January 2010 for King George island samples (Carrasco et al., 2012); January 2014 for Deception, Snow, Dee, Livingstone, Greenwich, Robert, Nelson and Litchfield islands (Troncoso et al., 2017); November 2014 for Union Glacier (Barahona et al., 2016); and January 2015 for Lagotellerie island. The geographical distribution of islands and Union Glacier is shown in **Figure 1**, including detailed collection sites from Lagotellerie. The details of the collection sites of other islands and Union Glacier were previously described (Carrasco et al., 2012; Barahona et al., 2016; Troncoso et al., 2017).

### DNA Extraction, PCR Amplification and Sequencing

A PowerSoil^®^ DNA Isolation Kit (MO BIO Laboratories Inc., Carlsbad, CA, United States) was used for direct DNA extraction from soil samples. The manufacturers’ instructions were followed, with only one modification, the disruption step, was performed using a Mini Beadbeater-16 cell disrupter (BioSpec Bartlesville, United States) instead of vortex agitation, as no PCR-amplicons were obtained in reactions using samples obtained through vortex agitation. The PCR reactions were performed using 1 μl of DNA sample (direct or 1/10 dilution), 5 units of *Taq* DNA polymerase, dNTP mix at 0.4 mM each, forward and reverse primers at 1 mM final each, PCR buffer and MgCl_2_ 2 mM. Amplification was performed using a 2720 (Applied Biosystems) thermal cycler using the following protocol: initial denaturation at 94°C for 3 min; 35 cycles of denaturation at 94°C for 30 s, annealing at 50°C for 3 min, and extension at 72°C for 3 min; and a final extension step at 72°C for 10 min. The universal primers F63 (5′-GCA TAT CAA TAA GCG GAG GAA AAG-3′) and LR3 (5′-GGT CCG TGT TTC AAG ACG G-3′) were used; these primers have been described as specific for amplifying the fungal D1/D2 region of the large subunit ribosomal gene (LSU) (Fell et al., 2000). In all PCR reactions, the same forward primer (F63) was used, but the reverse primer (LR3) was specific for each soil sample as a 454 adapter, a specific barcode and a linker sequence were added. Sequencing was performed at OMICS Solutions (Santiago, Chile) using the Ion 314^TM^ Chip Kit v2 (Thermo Fisher) and Ion Torrent personal genome machine (PGM), according to the manufacturer’s instructions (Rothberg et al., 2011). Three independent runs were performed: (i) samples from King George Island; (ii) samples from Deception, Snow, Dee, Livingstone, Greenwich, Robert, Nelson and Litchfield islands; and iii) samples from Lagotellerie and Union Glacier. Sequence data were deposited at the NCBI: Bioprojects PRJNA395715 and PRJNA397058.

### Bioinformatics Sequence Analysis

Sequences were analyzed using Geneious 10 software and its plugins. Chimera sequences were removed using UCHIME v4.2.40 (Edgar et al., 2011). Sequences were classified according to the collection site where they were obtained according to barcodes, and primer sequences were removed. Sequences were compared, and those having ≥ 97% identity were clustered into an operational taxonomic unit (OTU); sequences that were not grouped into OTU were classified as Unique Sequences (UnSeqs). For comparative purposes, the OTU were generated considering the data independently at three geographical levels: (i) “Site,” considering sequences from each collection site; (ii) “Locality,” considering sequences from all collection sites on an island or in Union Glacier; and iii) “All,” considering the sequences from all collection sites obtained in this work. The OTU and UnSeq number and length average are shown in **Supplementary Figure S1**. OTU with a sequence length of at least 300 nt were compared against the NCBI database using nucleotide BLAST (BLASTn). Sequences displaying BLAST-hits of at least 90% identity and 70% coverage were considered for tentative OTU annotation. In the first instance, the search was filtered excluding sequences from uncultured sources at the NCBI database; then, sequences not annotated this way were compared again against the NCBI database, but this time considering sequences from uncultured sources. The majority of OTU displaying BLAST-hits had sequence lengths between 500 and 550 nt and showed a high percentage of identity and coverage to its best BLAST-hit.

### Isolation and Identification of Yeasts

Five grams of each soil or sedimentary rock sample was mixed with 6 ml of sterile water and agitated in a vortex for 16 h at 22°C using an Intelli Mixer RM-2M (ELMI, Calabasas, CA, United States). After decantation of coarse particulate material, 200 μl aliquots of soil suspension were seeded onto YM agar plates (0.3% yeast extract, 0.3% malt extract, 0.5% peptone, 1.5% agar) supplemented with 1% glucose or 1% maltose and onto MYP agar plates (0.7% malt extract, 0.5% yeast extract, 2.5% peptone, 1.5% agar, pH 5.0). All plates were supplemented with 100 μg/ml of both ampicillin and chloramphenicol. Three plate replicates were incubated at 15°C and 22°C and periodically inspected. Once yeast-like colonies were observed, they were transferred to a new plate with fresh media. For yeast DNA extraction, a loopful of yeast from agar plates was suspended in 600 μl of TE buffer (Tris 25 mM, EDTA 10 mM, pH 8.0) and mixed with 100 μl of 0.5-mm diameter glass beads and 600 μl of phenol:chloroform:isoamyl alcohol (25:24:1, v/v). Cell disruption was performed using a Mini-Beadbeater-16 cell disrupter (BioSpec Products Inc., Bartlesville, OK, United States) for 30 s. Samples were centrifuged at 10,000 × *g* for 10 min, and the supernatant was transferred to a new tube where it was mixed with 1 ml cold ethanol and incubated for 2 h at -20°C. After centrifugation at 10,000 × *g* for 10 min, the obtained DNA pellet was washed with 70% ethanol, dried at 37°C for 10 min and suspended in 100 μl of TE buffer with 40 μg/ml of RNase A. The integrity and concentration of the DNA samples were checked by 1.5% agarose gel electrophoresis, using as a reference a 1-Kb DNA ladder (New England Biolabs, Ipswich, MA, United States). From DNA samples, the D1/D2 region of the 18S rDNA was PCR-amplified using the universal primers F63 and LR3 (Fell et al., 2000). Amplicons were resolved via 1.5% agarose gel electrophoresis and purified from gels (Boyle and Lew, 1995), and the nucleotide sequences were determined using the sequencing services of Macrogen Inc. (Seoul, Korea). Sequences were analyzed using Geneious Pro 10 software (Biomatters, Auckland, New Zealand), and taxonomic analysis was performed using the maximum likelihood method based on the Tamura–Nei model (Tamura and Nei, 1993) conducted with MEGA7 software (Kumar et al., 2016). Only sequences from yeast type species from the taxonomic revision of Basidiomycota were used as references (Wang et al., 2015a,b), which were downloaded from the NCBI database.

## Results

### Localities at Similar Latitude Are More Similar at Molecular Level

**FIGURE 2:**
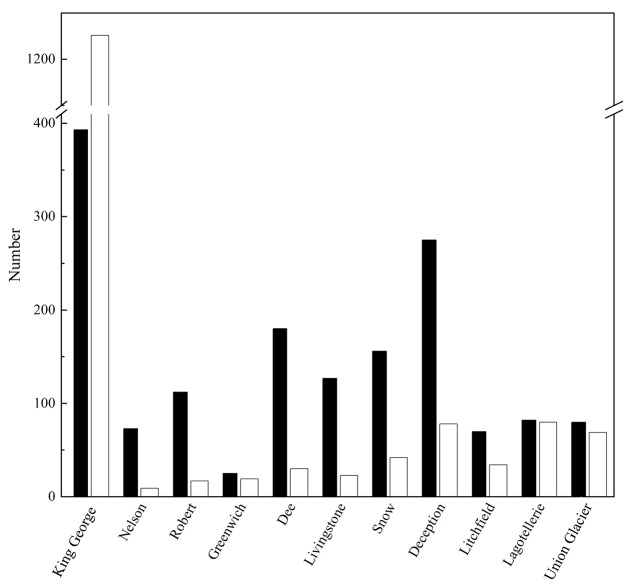
Shared and exclusive OTU of each Locality. Number of OTU in a Locality also present in at least one other Locality (black columns), and those exclusively present in the corresponding Locality (white columns), are shown.

**FIGURE 3:**
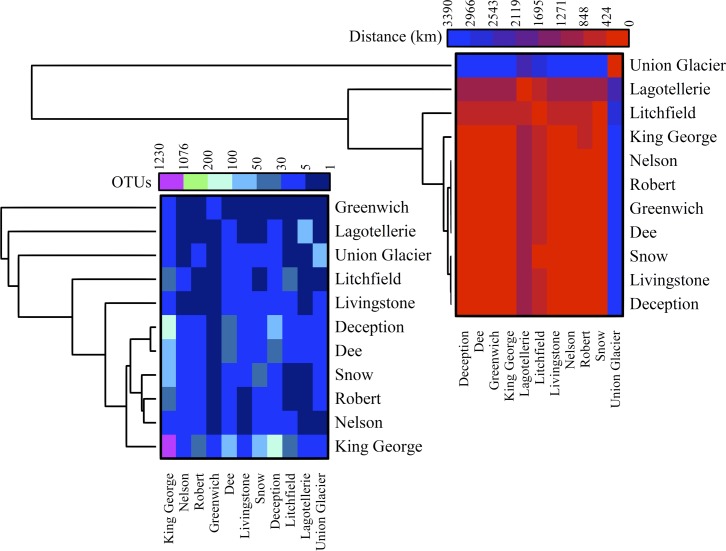
Cross-study comparisons among Localities. All localities were compared based on number of shared OTU **(left)** and geographic distance between them **(right)**. For each case, hierarchical clustering combined with heat-map is shown. Scales are indicated at top of each heat-map and black indicates no BLAST-hits.

**FIGURE 4:**
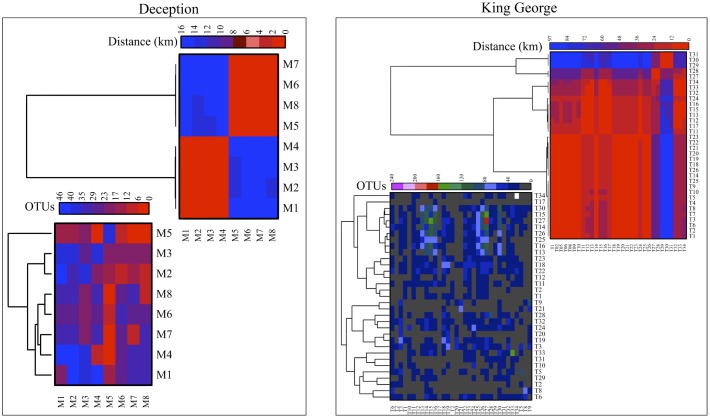
Cross-study comparisons among sampling Sites. All sampling sites were compared based on number of shared OTU **(left)** and geographic distance between them **(right)**. For each case, hierarchical clustering combined with heat-map is shown. Scales are indicated at top of each heat-map and black indicates no BLAST-hits.

The number of OTU and UnSeq sequences generated were similar when considering all data, and the length of most of them ranged from 500 to 550 nt (**Supplementary Figure S1A**). This was similar at the Locality and Site levels (**Supplementary Figures S1B,C**), with the exception of sequences from King George Island, where a high number of sequences were obtained but with shorter average length. Considering the OTU generated with all data, the sequences were classified as “shared” or “exclusive” if they were found in at least two Localities or a single Locality, respectively. Except for King George Island, the Localities had more shared than exclusive OTU (**Figure 2**). Higher percentages of shared OTU were found in Nelson (89%), Robert (87%) and Dee (86%) islands. The Localities that shared the highest number of OTU were Deception and King George (101 OTU), Dee and King George (71 OTU), Robert and King George (37 OTU), and Dee and Deception (36 OTU). Considering the distance between these Localities—Deception and King George (154 km), Dee and King George (83 km), Robert and King George (64 km), and Dee and Deception (73 km)—there is no correlation between the geographic distance among Localities and “molecular” similarity. To test this preliminary observation, all Localities were compared and hierarchically grouped according to geographic distance and molecular similarity. As shown in **Figure 3**, molecular clustering grouped the islands of the South Shetland Archipelago, except Greenwich Island, and separated them from Litchfield and Lagotellerie islands and Union Glacier, in agreement with their geographical clustering. No correspondence was observed between molecular and geographic clustering among islands from the South Shetland Archipelago, e.g., at the molecular level, Nelson and Robert islands (being both the most geographically closer) grouped with Snow island but not Greenwich island, even though the latter is closer to them. Similar results were observed when the collection Sites in a Locality were analyzed. As an example, there was no coincidence between the molecular and geographic clustering of two groups of samples (M1–M4 and M5–M8) collected from different extremes of Deception island, which were separated by Neptune’s Bellows channel (a channel on the southeast side forming the entrance to Port Foster). Another example is the T2 and T29 sampling sites from King George Island, which grouped together in the molecular analysis even though they are geographically further away than other King George’s sampling sites; T2 is located at the Fildes Peninsula and T29 at the Lions Rump (45 km apart) (**Figure 4**). Similar results were observed when other sampling sites from other islands or sites at Union Glacier were analyzed (**Supplementary Figure S2**). Considering these results, a correspondence to molecular similarity can be observed that accounts for the latitude of Localities studied.

### Fungal Species Distribution in Antarctic Localities

**FIGURE 5:**
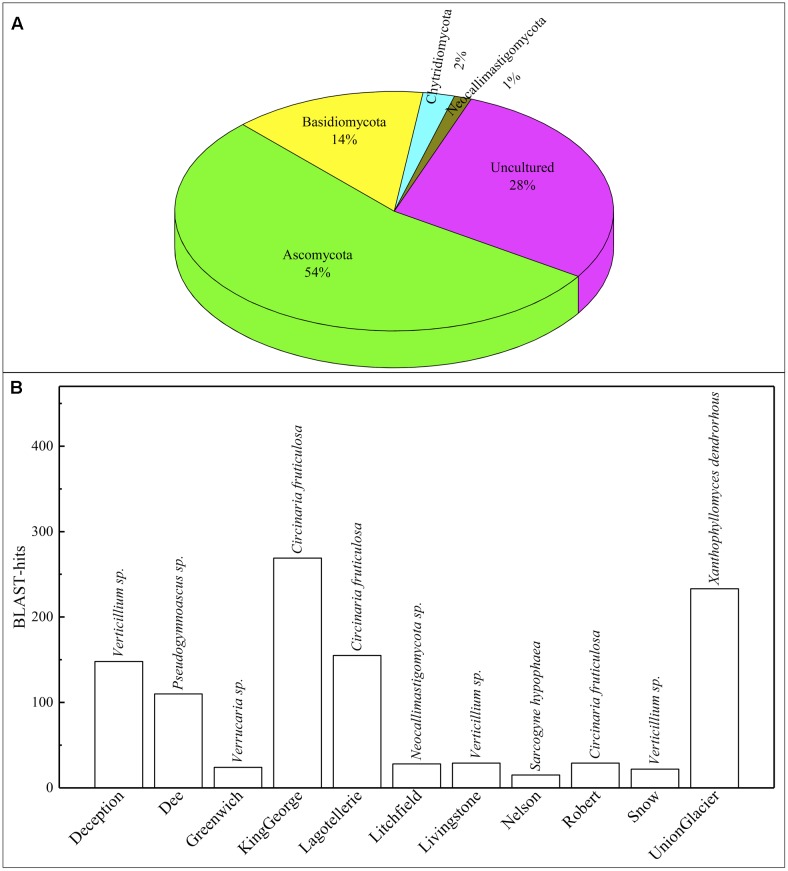
Operational taxonomic unit (OTU) comparisons against NCBI database. **(A)** Distribution of OTU BLAST-hits according to sources. **(B)** Distribution of BLAST-hits from species. Species with the most BLAST-hits is indicated on the top of column.

**FIGURE 6:**
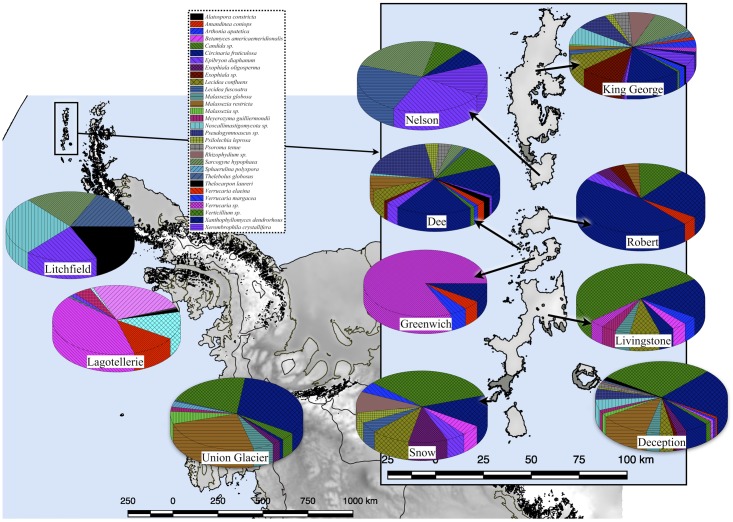
Fungal species distribution among Localities. Only results for fungi found in at least 4 Localities are shown.

A 54 and 14% of fungal sequence BLAST-hits belonged to the Ascomycota and Basidiomycota phyla, respectively (**Figure 5A**), of which almost half (47%) corresponded to lichenized fungi. If only BLAST-hits from yeasts sources were considered, a 68% were basidiomycetes. The higher number of OTU sequences with BLAST-hits obtained at the species level were from King George island and Union Glacier, in which the major species corresponded to *Circinaria fruticulosa* and *Xanthophyllomyces dendrorhous*, respectively (**Figure 5B**). The fungal BLAST-hits were distributed in 87 genera and 123 species, of which the highest numbers were found for the genera *Xanthophyllomyces*, *Circinaria*, *Verticillium*, *Malassezia* and *Verrucaria* (**Supplementary Figure S3A**) and the species *X. dendrorhous*, *C. fruticulosa*, *Verticillium* sp., *Malassezia restricta*, *Pseudogymnoascus* sp. and *Thelebolus globosus*. The Sites with the highest number of fungal BLAST-hits were Lagotellerie I7, Union Glacier T9, Union Glacier T7, Dee M14, Dee M15, Union Glacier T13 and Deception M1 (**Supplementary Figure S3B**). Considering the number of different putative fungal species, the highest number was found at King George (73 species), Deception (33 species), Dee (28 species) and Lagotellerie (22 species) Islands. The most ubiquitous species and Localities with the highest number of BLAST-hits obtained were *X. dendrorhous* (10 localities/Union Glacier), *Verticillium* sp. (7 localities/Union Glacier), *C. fruticulosa* (7 localities/Lagotellerie), *M. restricta* (6 localities/Union Glacier), *T. globosus* (6 localities/Lagotellerie) and *Epibryon diaphanum* (6 localities/Dee) (**Figure 6**). Primers were designed based on the OTU sequences of *X. dendrorhous* and *Malassezia* species, for which a high number of BLAST-hits were obtained, especially from King George Island and Union Glacier, to perform PCR reactions with newly purified DNA from soil samples from different localities using new DNA extraction kits, reagents, and enzymes; these were performed under a laminar flow chamber. Amplicons of the expected lengths were obtained from the DNA samples of these different localities, which were not obtained in controls with no DNA (Supplementary Table S1), supporting the results obtained in the amplicon-metagenome analysis.

**FIGURE 7:**
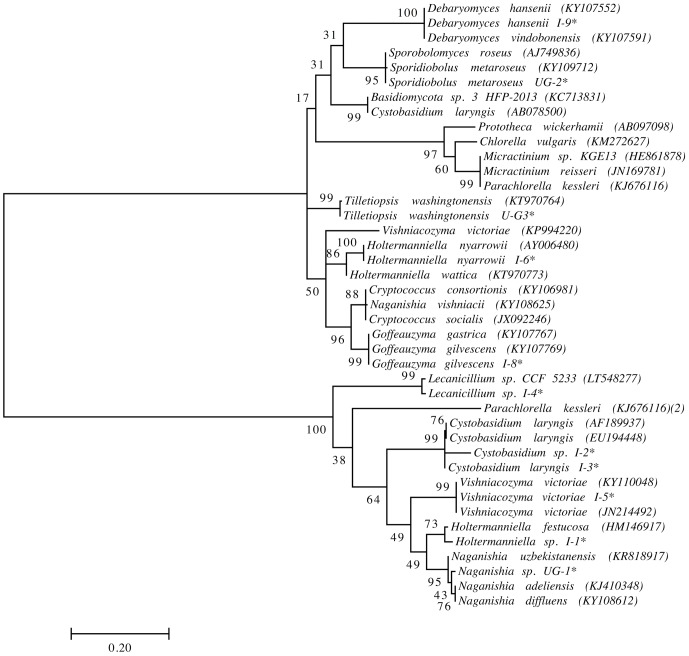
Phylogenetic placement of yeast isolates. Analysis was performed with D1/D2 domain sequences using the maximum likelihood method. Bootstrap values higher than 50% are shown (1,000 replicates). ^∗^Sequences obtained in this work. Accession number of sequences obtained from the NCBI database in parentheses.

We previously isolated and identified yeast species from the same soil and sedimentary rock samples analyzed in this work (Carrasco et al., 2012; Contreras et al., 2015; Barahona et al., 2016; Troncoso et al., 2017). However, yeasts highly represented in this amplicon-metagenomic study such as *Xanthophyllomyces* and *Malassezia* species from the Lagotellerie island and Union Glacier have not been isolated. Therefore, we renewed our efforts to cultivate yeasts from samples from these two localities using different culture conditions. The yeasts isolated were identified according to the identity (≥99.5%; (Kurtzman and Fell, 2006)) of their D1D2 rDNA region sequence to the closest known yeast species and taxonomic position (**Figure 7** and Supplementary Table S2). In this way, we were able to isolate and identify yeast species belonging to the genera *Cystobasidium*, *Debaryomyces*, *Goffeauzyma*, *Sporidiobolus*, *Vishniacozyma* and *Holtermanniella*, which were previously identified by us (Carrasco et al., 2012; Barahona et al., 2016; Troncoso et al., 2017) and yeasts of genera *Lecanicillium*, *Naganishia* and *Tilletiopsis* that have not been previously isolated from these localities.

### Analysis of UnSeq and Comparison to Results from OTU

Taking into account the BLAST-hits, obtained for UnSeq sequences, with higher identity, coverage and ranged from 500 to 550 nt, a 57% corresponded to fungal sources, most of which belonged to the phyla Ascomycota (44%) and Basidiomycota (15%) (**Supplementary Figure S5A**). The Localities with the highest number of fungal BLAST-hits were King George Island and Union Glacier (**Supplementary Figure S5B**), and the Sites were Union Glacier T9, Greenwich M18 and Union Glacier T7. The most ubiquitous species were *Verticillium* sp., *X. dendrorhous* and *E. diaphanum*, which were found in 6, 6 and 5 Localities, respectively.

Thirty-six fungal genera were found in the analysis of both OTU and UnSeq sequences, while 47 and 46 genera were found only in OTU and UnSeq, respectively (**Supplementary Figure S6A**). Compared to the cultivable fungi described from Antarctica, only 24 fungal genera were detected in the OTU analysis, a number that increased to 36 if results from UnSeq were also considered (**Supplementary Figure S6A**). If the comparison is restricted to viable Antarctic yeast species described in the literature, only two and one were found in OTU and UnSeq analysis, respectively (**Supplementary Figure S6B**). Considering the cultivable yeasts isolated from the same samples by our group, only *Vishniacozyma victoriae* and *Goffeauzyma gastrica* were also found in the OTU and UnSeq analysis, respectively.

### Phylogenetic Analysis and Worldwide Distribution

**FIGURE 8:**
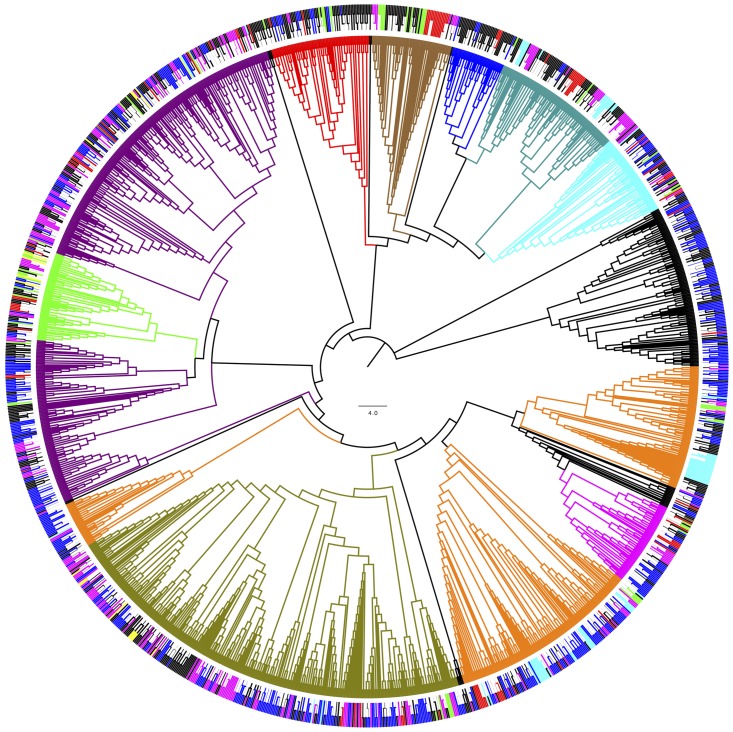
Phylogenetic grouping of the OTU fungal BLAST-hits. Bootstrap values higher than 50 % are shown (500 replicates). **(Inner)** Coloring according to fungal class: Agaricomycetes (Blue), Dothideomycetes (green), Chytridiomycetes (red), Eurotiomycetes (dark green) Lecanoromycetes (violet), Leotiomycetes (orange), Microbotryomycetes (cyan), Saccharomycetes (magenta) and Tremellomycetes (olive). **(Outer)** Coloring according to continent of origin: Africa (brown), Asia (red), Europa (magenta), North America (blue), Oceania (yellow), South America (green). ^∗^Uncultured fungal sources. OTU obtained in this work are shown in black.

**FIGURE 9:**
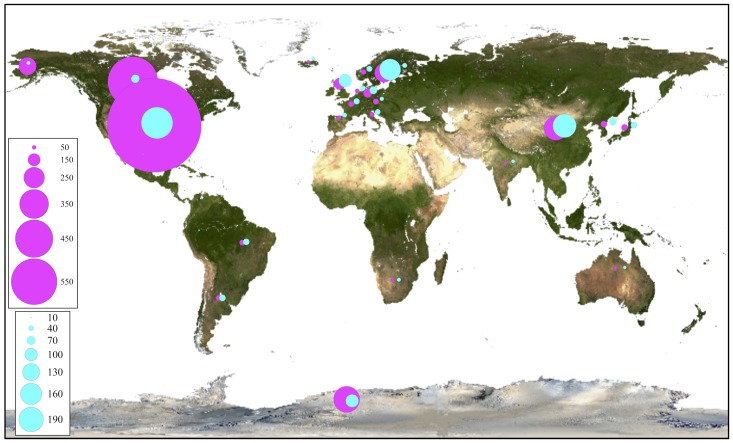
Global distribution of closest OTU fungal BLAST-hits. All BLAST-hits (magenta) and BLAST-hits excluding those from uncultured sources (cyan), are represented. Scales indicate number of BLAST hits.

For phylogenetic analysis, only the fungal OTU having sequence length ≥ 500 nt were considered together with their closest BLAST-hit, which were clustered into 11 classes (**Figure 8**). Each class contained sequences reported from countries on several continents, with the exception of one class that included uncultured fungal sequences reported mainly from northeast San Diego County, CA, United States and other localities in North America. The most represented fungal classes were Lecanoromycetes (sequences from 36 countries, mainly from Sweden) and Eurotiomycetes (sequences from 27 countries, mainly from the United Kingdom). The Agaricomycetes (sequences from 9 countries) and Microbotryomycetes (sequences from 12 countries, mainly from the United States) were less represented fungal classes. In relation to non-fungal eukaryotes, the most represented classes were Bryopsida, Ascochloris and Bacillariophyceae (**Supplementary Figure S7**). Unlike fungal sources, the geographic origin for most non-fungal BLAST-hits sequences was not available. However, the few sequences with this information available come from several continents. The global distribution of fungal BLAST-hits and OTU sequences is shown in **Figure 9**. Most uncultured fungal sources were reported in the United States and Canada, and when only cultured fungi were considered, most of them were reported in the United States, China, and Nordic countries.

## Discussion

A correlation between micro-eukaryotic diversity and distance and geographic barriers of the Antarctic localities studied in this work was observed, but only at large geographical scale finding a major molecular difference between the islands of the South Shetland archipelago (latitude S61–S63) and the localities on the Antarctic peninsula (S64–S80). However, as in any ecosystem, other abiotic and biotic factors not considered in this study may influence microbial communities. Similar to our results, Lawley et al. (2004) found that the hypothesis of an inverse relationship between eukaryotic diversity and latitude was valid only in a large-scale comparison and only when maritime and continental diversity were compared. Also, an inverse relationship between bacterial diversity and latitude was observed in soils from the Falkland Islands (51°S) to the Ellsworth Mountains (78°S), but only in fell-field soils, not vegetated sites (Yergeau et al., 2007). In other studies, soil pH was proposed as an important factor for structuring bacterial assemblage patterns (Chong et al., 2010, 2012).

Even though the present work was focused on fungal diversity using the primers LR3 and F63 (Fell et al., 2000), a 36% of the obtained sequences corresponded to green algae and diatoms found in all localities studied (**Supplementary Figures S4**, **S5A,C**). Therefore, in environmental culture-independent studies, it should be considered that the primers LR3 and F63 may also amplify non-fungal DNAs. Similarly, in studies based in amplification of fungal ITS2 region in the Antarctic active layer, a high number of sequences identified as mosses, green algae and infusoria were previously found (Kochkina et al., 2014). The putative algae and diatom species found in Antarctic soils in this work have been described as ubiquitous environmental species. *S. bacillaris* is a common terrestrial alga, with a cosmopolitan distribution that was previously described at Antarctic and Sub-Antarctic islands (Broady, 1979). The diatom *H. amphioxys* is a freshwater/terrestrial species that has been described worldwide, including saline lakes in the Larsemann Hills and Rauer Islands east of Antarctica (Sabbe et al., 2003). The diatom *P. taeniata* has been described as a marine species from Europe, Asia, North America and the Arctic sea (Brown et al., 2014), but to the best of our knowledge, it has not been previously described in Antarctica.

Most fungi detected in this work were ascomycetes, in accordance with literature referring to terrestrial fungi from Antarctica (Arenz et al., 2014). Putative species belonging to 37 fungal genera were detected in this work that has not been previously described from Antarctica. It has been suggested that the majority of non-lichenized fungal species described in Antarctica are cosmopolitan groups (Bridge and Spooner, 2012). In this work, almost half of the fungi (excluding yeasts) identified corresponded to non-lichenized fungi, whose closest BLAST-hits were described in several countries, primarily the USA, China and Argentina. However, the closest BLAST-hits for lichenized fungi were described in several countries, primarily Sweden, United States and the United Kingdom. In contrast, 68% of the yeasts detected in this work were basidiomycetes, in agreement with our previous work on cultivable yeasts from these soils (74% corresponded to basidiomycetes) (Carrasco et al., 2012; Contreras et al., 2015; Barahona et al., 2016; Troncoso et al., 2017) and other and other studies from Antarctic terrestrial habitats (84% basidiomycetes) (Buzzini et al., 2012; Selbmann et al., 2014; Vasileva-Tonkova et al., 2014; Zhang et al., 2014; Duarte et al., 2016; Martinez et al., 2016). The predominance of the yeast phylum Basidiomycota has been attributed to their ability to produce polysaccharide capsules, utilize the available nutrients in oligotrophic systems and increase the proportion of unsaturated fatty acids (Connell et al., 2008; Margesin and Miteva, 2011; Buzzini et al., 2012).

## Conclusion

Among the fungal sequences detected by the amplicon-metagenomic approach used in this work, thirty-seven corresponded to genera not previously cultivated from Antarctica. The most represented fungal classes were Lecanoromycetes and Eurotiomycetes. Considering the rDNA sequences similarities and the geographical positioning of the localities studied, a correlation between microbial communities and geographic latitude was observed.

## Availability of Data and Material

The datasets generated and/or analyzed during the current study are available in the NCBI database: Bioprojects PRJNA395715 and PRJNA397058.

## Author Contributions

MB conceived the study. SB performed DNA extraction from soils, PCR reactions, and isolation and identification of yeasts. MB and JA analyzed the results. MB, JA, and VC wrote the manuscript. All authors read and approved of the final manuscript.

## Conflict of Interest Statement

The authors declare that the research was conducted in the absence of any commercial or financial relationships that could be construed as a potential conflict of interest.

## Supplementary Material

**FIGURE S1 |** Operational taxonomic unit and UnSeq generated from soil samples. Analysis was performed considering the data from each Site **(A)**, Locality **(B)**, and All data **(C)**. Closed circles and closed squares correspond to number and average length of OTU, respectively. Open circles and open squares correspond to number and average length of UnSeqs, respectively.

**FIGURE S2 |** Cross-study comparisons among Sites. All Sites were compared based on their shared OTU and geographical distances between them. Hierarchical clustering combined with heat mapping shown for distance and OTU. Scales are indicated at top of each heat map.

**FIGURE S3 |** Distribution of fungal BLAST-hits. Heat-map of number of fungal BLAST-hits (at the genus level) distributed by Localities **(A)** and collection Sites **(B)**. Color scale at right of each heat-map indicates number of BLAST-hits and black indicates no BLAST-hits.

**FIGURE S4 |** Distribution of non-fungal BLAST-hits. Heat-map of number of non-fungal BLAST-hits (at the genus level) distributed by Localities **(A)** and collection Sites **(B)**. Color scales indicate number of BLAST hits and black indicates no BLAST-hits.

**FIGURE S5 |** Comparison of UnSeq against NCBI database. **(A)** Distribution of BLAST-hits among phyla. Number of fungal **(B)** and non-fungal **(C)** BLAST-hits at genus level distributed by Localities. Color scales indicate number of BLAST hits and black indicates no BLAST-hits.

**FIGURE S6 |** Comparison of fungi identified from OTU and UnSeq sequences, and cultivable fungal genera described from Antarctica. **(A)**, Fungal genera; **(B)**, yeast species; ^∗^cultivable yeasts isolated by our group from same samples used in the present amplicon-metagenomic analysis. ^∗∗^Fungal genus detected in this work that have not previously described from Antarctica.

**FIGURE S7 |** Phylogenetic grouping of OTU of non-fungal BLAST-hits. Bootstrap values higher than 50 % are shown (500 replicates). Inner, coloring according to class: Andreaeopsida (Blue), Bryopsida (green), Bacillariophyceae (red), Nephroselmidophyceae (yellow), Ascochloris (violet), Acrispumella (orange), Trebouxiophyceae (cyan), Spirotrichea (magenta) and Ulvophyceae (brown). Outer, coloring according to continent of origin: Africa (brown), Asia (red), Europa (magenta), North America (blue), Oceania (yellow), South America (green). OTU obtained in this work are shown in black.
