# Supplementary material for: Amplicon-Metagenomic Analysis of Fungi from Antarctic Terrestrial Habitats
Source: Front Microbiol. 2017 Nov 14;8:2235. doi: 10.3389/fmicb.2017.02235 (PMC5694453; doi:10.3389/fmicb.2017.02235)
Supplement: Supplementary file 8 [file Table_1.PDF]

Table S1. PCR results from soil samples with primers specific for *Xanthophyllomyces* and *Malassezia* genera.

	Malassezia				*Xanthophyllomyces*			
	Ma43F/Ma394R (351)	Ma43F/Ma497R (454)	Ma70F/Ma394R (324)	Ma70F/Ma497R (427)	XD18F/XD347R (329)	XD18F/XD411R (393)	XD27F/XD347R (320)	XD27F/XD411R (384)
Decepcion	-	+	+	+	-	+	+	-
Dee	-	+	+	+	+	+	+	-
Greenwich	+	+	+	+	+	+	+	+
King George*	+	+	+	+	+	+	+	+
Lago Tellerie	+	+	+	+	+	+	+	+
Litchfield	+	+	+	+	+	+	+	+
Livingstone	-	+	+	+	-	+	+	-
Nelson	+	+	+	+	+	+	+	+
Robert	-	+	+	+	-	+	+	-
Snow	-	+	+	+	-	+	+	-
Union Glacier*	-	-	+	+	+	+	+	-

In parenthesis is indicated the length of the expected amplicon for each primers pair. *, PCR reactions were perform with newly purified DNA

Primer sequences

**Primer**	**Sequence, 5'->3'**	**Primer**	**Sequence, 5'->3'**	**Primer**	**Sequence, 5'->3'**	**Primer**	**Sequence, 5'->3'**
Ma43F	gAACAgggCATCgTAgAggg	Ma394R	AggATgCCACATTCCCCATg	Xd18F	TATgCCAgCATCCTAAgCgC	Xd347R	ATTggCgAgAgACCgATAgC
Ma70F	CCCgTACTTgCCATggAAgT	Ma497R	CgCCAgCATCCTAAgTgTgA	Xd27F	ATCCTAAgCgCgTTCgTgTC	Xd411R	ACgAgTCgAgTTgTTTgggA
