# Supplementary material for: Amplicon-Metagenomic Analysis of Fungi from Antarctic Terrestrial Habitats
Source: Front Microbiol. 2017 Nov 14;8:2235. doi: 10.3389/fmicb.2017.02235 (PMC5694453; doi:10.3389/fmicb.2017.02235)
Supplement: Supplementary file 9 [file Table_2.PDF]

Table S2. Molecular identification of yeasts isolated from Lagotellerie island and Union Glacier.

Isolates	NCBI	Closest BLAS-THits	Locality	O; I; G	Proposed Identification
I1-22, I1-1-22 and I3-22	KY363485	*Cystobasidium laryngis* (EU194448)	Lagotellerie	610; 99.8; 0	*Cystobasidium laryngis* I-3
I1-22-A(15 A)	KY363486	*Cystobasidium laryngis* (AF189937)	Lagotellerie	521; 92; 11	*Cystobasidium* sp. I-2
I4-30 and I4-2-30	KY363487	*Debaryomyces hansenii* (KY107552)	Lagotellerie	585; 99.8;1	*Debaryomyces hansenii* I-9
I4-22, I4-10 and I4-4	KY363488	*Goffeauzyma gilvescens* (KY107769)	Lagotellerie	557; 100;0	*Goffeauzyma gilvescens* I-8
I3-10	KY363489	*Holtermanniella nyarrowii* (AY006480)	Lagotellerie	598; 100; 0	*Holtermanniella yarrowii* I-6
I1-10, I1-1-4 and I3-4	KY363490	*Holtermanniella festucosa* (HM146917)	Lagotellerie	599;98.4; 0	*Holtermanniella* sp. I-1
I1-4, I1-30 and I6-22	KY363491	*Lecanicillium* sp. CCF 5233 (LT548277)	Lagotellerie	581; 98.8; 2	*Lecanicillium* sp. I-4
GUT2-2, GUT10-1, GUT10-2, GUT10-3, GUT10-4, GUT10-5, GuT10-6 and GUT10-6-3	KY363493	*Naganishia uzbekistanensis* (KR818917)	Union Glacier	621; 98.1; 2	*Naganishia* sp. UG-1
GUT1-5, GUT2-1, GUT3 GUT4, GUT4 GUT6 and GUT8	KY363494	*Sporidiobolus metaroseus* (KY109712)	Union Glacier	588; 100; 0	*Sporidiobolus metaroseus* UG-2
GUT1-8	KY363495	*Tilletiopsis washingtonensis* (KT970764)	Union Glacier	610; 100; 0	*Tilletiopsis washingtonensis* U-G3
I2-4, I2-1-4 and I2-2-4	KY363492	*Vishniacozyma victoriae* (KY110048)	Lagotellerie	565; 99.5; 0	*Vishniacozyma victoriae* I-5

O, nucleotide overlap; I, % of identity; G, number of gaps. Accession numbers of BLAST- hits are given in parenthesis.
